# Macular Ganglion Cell Complex and Peripapillary Retinal Nerve Fiber Layer Thinning in Patients with Type-1 Gaucher Disease

**DOI:** 10.3390/ijms21197027

**Published:** 2020-09-24

**Authors:** Yishay Weill, Ari Zimran, David Zadok, Lauren M. Wasser, Shoshana Revel-Vilk, Joel Hanhart, Tama Dinur, David Arkadir, Michal Becker-Cohen

**Affiliations:** 1 Department of Ophthalmology, Shaare Zedek Medical Center, Jerusalem 9103102, Israel; davzadok@gmail.com (D.Z.); lauriew21@gmail.com (L.M.W.); hanhart@szmc.org.il (J.H.); 2 Faculty of Medicine, The Hebrew University, Jerusalem 9112001, Israel; azimran@gmail.com (A.Z.); srevelvilk@gmail.com (S.R.-V.); dinurtama@gmail.com (T.D.); arkadir@gmail.com (D.A.); michalbec@gmail.com (M.B.-C.); 3 Gaucher Unit, Shaare Zedek Medical Center, Jerusalem 9103102, Israel; 4 Department of Neurology, Hadassah Medical Center, Jerusalem 9112001, Israel

**Keywords:** Gaucher disease, retinal thinning, optical coherence tomography, retinal nerve fiber layer, retinal ganglion cells, ganglion cell complex, neurodegenerative disorder

## Abstract

Type-1 Gaucher disease (GD1) is considered to be non- neuronopathic however recent evidence of neurological involvement continues to accumulate. There is limited evidence of retinal abnormalities in GD1. The purpose of this study was to evaluate the retinal findings of patients with GD1. Thirty GD1 individuals and 30 healthy volunteers between the ages 40–75 years were prospectively enrolled. Macular and optic nerve optical coherence tomography (OCT) scans of both eyes of each patient were performed and thickness maps were compared between groups. Patients with a known neurodegenerative disease, glaucoma, high myopia and previous intraocular surgeries were excluded. It was shown that patients with GD1 presented with higher incidence of abnormal pRNFL OCT scan and showed significantly thinner areas of pRNFL and macular ganglion cell complex (GCC) when compared to a healthy control population. Changes in retinal thickness were not associated with GD1 genotype, treatment status, disease monitoring biomarker (lyso-Gb1) and severity score index (Zimran SSI). Further investigations are needed to determine whether these findings possess functional visual implications and if retinal thinning may serve as biomarker for the development of future neurodegenerative disease in this population.

## 1. Introduction

Gaucher disease (GD), although rare, is one of the most common inherited lysosomal storage disorders [1,2]. It results from an autosomal recessive mutation in the GBA gene which encodes the glucocerebrosidase enzyme. The decrease in enzymatic activity leads to glucosylceramide accumulation in macrophage lysosomes. Traditionally, GD is classified into three clinical presentations. Type 1 (adult, chronic non-neuronopathic) is defined by the absence of neurological features (symptomatic patients usually present with hepatosplenomegaly and bleeding tendency and may develop various skeletal abnormalities if untreated). Type 2 (infantile, acute neuronopathic) is defined by early development of severe neurological abnormalities and typically leads to death by the age two. Type 3 (juvenile, subacute neuronopathic) is defined by the existences of less severe and more variable central nervous system involvement when compared to type 2. Typical neurological features in type 3 include supranuclear gaze palsy and myoclonic epilepsy [2,3]. Of the three types of GD, type-1 (GD1) is the most common, representing over 90% of all cases in the western hemisphere and is historically differentiated from other GD types by the lack of central nervous system involvement [2,3]. Nevertheless, neurological involvement, such as Parkinson disease (PD), cognitive impairment and dementia, have been reported over the last two decades in patients with GD1 [4,5,6,7,8]. Moreover, histopathological studies of the brain of symptomatic and asymptomatic patients with GD1 identified unique cortical pathologic pattern [9]. These findings have led Sidarsky et al. and other investigators to challenge the common GD classification of neurological involvement and to propose that GD should be perceived as a phenotypic continuum [4,6,10].

**Figure 1:**
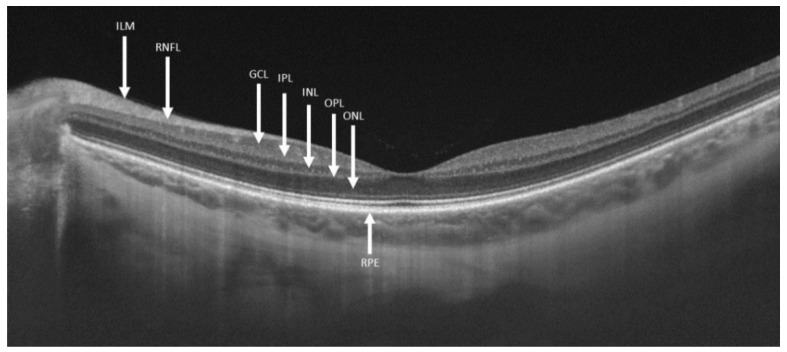
Segmentations of retinal layers in swept-source optical coherence tomography. Abbreviations: ILM—inner limiting membrane; RNFL—retinal nerve fiber layer; GCL—ganglion cell layer; IPL—inner plexiform layer; INL—inner nuclear layer; OPL—outer plexiform layer; ONL—outer nuclear layer; RPE—retinal pigmented epithelium.

The human retina, an extension of the central nervous system, is a light-sensitive neuronal tissue that consists of nine layers of cells that together convert light rays into a neuronal impulse. The visual information is produced by the photoreceptors and spread to the retinal ganglion cells (RGC) via bipolar cells. The RGC somas reside in the ganglion cell layer (GCL) in the inner retina and it is their axons that form the retinal nerve fiber layer (RNFL) which comprise the optic nerve (ON). From the ON the visual information is transmitted via axons leading to the lateral geniculate body [11]. Damage to RGC along their course can be expressed as cell body or axon injury. Optical coherence tomography (OCT) is a widely available noninvasive imaging technique used to evaluate the retina. This technique utilizes light waves to create multiple cross-sections of the retina providing high-resolution images (Figure 1). Among its various applications, OCT scans can measure the thickness of retinal layers in different areas, specifically the ON and macula [12].

While ocular symptoms may present as manifestations of GD, they are primarily observed in type-3 Gaucher disease (GD3), a neuronopathic form of GD [13]. Previous studies regarding ocular involvement in patients with GD1 consist mainly of small case studies [13]. Furthermore, macular GCL thickness and peripapillary RNFL (pRNFL) thickness as measured by OCT has not been systematically studied in patients with GD and previous reports have demonstrated conflicting results [14,15]. The goal of this study was to prospectively evaluate macular and peripapillary thickness utilizing OCT in a cohort of patients with GD1 compared to healthy controls.

## 2. Results

### Main Results

Differences in age, gender and refraction between patients with GD1 and controls were not significant (Table 1). Scan quality (ON and macula) did not differ between groups. Twenty-two eyes (36.7%) of patients with GD1 demonstrated abnormal pRNFL scans compared to 5 eyes (8.3%) in the control group (*P* < 0.01). Significant pRNFL and macular ganglion cell complex (GCC) thinning was observed in different ON quadrants (average RNFL, superior RNFL, inferior RNFL) and ETDRS sectors (outer nasal GCL, outer inferior GCL), respectively (Table 2). Other retinal parameters did not show significant change between the two groups (Supplemental data). No correlation was found in patients with GD1 between the average RNFL, superior RNFL, inferior RNFL, outer nasal GCL and outer inferior RNFL thickness and genotype (mild/severe), treatment status, lyso-Gb1 levels and Zimran SSI (Supplemental data).

## 3. Discussion

Evidence of neurological abnormalities in patients with GD1 has continued to accumulate, challenging the traditional classification of GD to neuropathic and non-neuronopathic types [10]. Since the retina serves as a “window” to the central nervous system and can be easily evaluated noninvasively, it is a commonly investigated tissue in many neurological disorders, frequently with the use of OCT. In this prospective study, we describe new retinal findings in patients with GD1. Patients with GD1 demonstrated significant macular GCC and pRNFL thinning. The observed thinning did not correlate with GD genotype, disease severity or treatment status. This study adds to the limited knowledge regarding retinal thickness in patients with GD, and to the best of our knowledge is the first study to evaluate RNFL thickness in patients with GD1.

McNeil et al. evaluated the macular GCC thickness in GD1 [14]. They examined the average GCC thickness in 11 patients with GD1 and three GBA mutation carriers. McNeil et al. separated the cohort into two groups depending on the presences or absence of clinical markers of a potential early neurodegenerative disorder (ND). They found significant thinning of the average macular GCC in the subgroup of four patients with GD1 and two GBA carriers with clinical markers of a potential early ND. However, in the subgroup of patients without such markers (seven patients with GD1 and one GBA carrier), average macular GCC thickness was similar to the healthy control group. McNeil et al. concluded that average macular GCC thinning may serve as a biomarker of increased risk of developing ND in patients with GD1.The findings of the present study are consistent with those of McNeil et al. as no significant changes were found in the average GCC thickness of patients with GD1. However, further stratification of the macula into subsections (as in the ETDRS) showed significant thinning of the outer macular GCC, more specifically in the outer nasal and outer inferior sections. As we excluded individuals with known ND and movement disorders in our study, these areas of focal thinning may potentially serve as an earlier biomarker for future development of ND in patients with GD1. It is important to note that in the ongoing follow-up of the GD1 cohort in our Gaucher unit (median 38 months, range 24 to 40 months), to date none of the patients had developed ND (specifically PD).

Retinal ganglion cells—whose axons form the ON—are metabolically active and sensitive to neurodegenerative damage due to ischemia, mitochondrial dysfunction, oxidative stress and abnormal axonal transport [11,16,17]. Mechanisms of RGC death include apoptosis and necrosis [18]. There are several mechanisms which may contribute to RGC impairment in patients with GD. First, systemic vascular accidents such as pulmonary hypertension and avascular necrosis as well as retinal vascular abnormalities such as occlusions, leakage and tortuosity, have been previously described in GD [13,19]. This implicates vascular damage as a cause of retinal ischemia and cell death in GD. Second, an increase in oxidative stress and impairment of the adaptive cellular response to oxidative stress, which have been documented in GD, may cause RGC injury [20,21]. Lastly, several reports suggest the findings of Gaucher cells in ocular tissues, specifically in the inner retina and ON [1,13]. Since the RGC nuclei are located in the inner retina and their axons comprise the ON, the presence of Gaucher cells in these locations may alter RGC function. Whether Gaucher cells negatively influence RGC or their supporting cells has yet to be evaluated.

More than 30 different subtypes of RGC have been described each possessing unique structure and visual function [22]. Individual RGC subtypes may be more metabolically active than others making them more vulnerable to cellular stress and cell death [11]. The small P-type RGC (midget cells), as an example, is responsible for transmitting high-quality colored visual information and require more energy than larger M-type cells (parasol cells), which transfer contrast and motion visual output [11,17]. Hence, P-type RGC are more susceptible to mitochondrial dysfunction [11]. Additionally, since some RGC types occupy a designated area of the retina, injury to specific cell types forms a unique pattern of RGC and RNFL loss. For instance, damage to P-type RGC will result in a temporal pRNFL loss (indicating papillo–macular bundle damage), while M-type RGC injury typically causes a vertical (superior and inferior) pattern of pRNFL loss [11,17]. In our study, we have demonstrated that patients with GD1 had a significant pRNFL thinning, specifically in the superior and inferior sections. This vertical pattern of pRNFL loss, with relative sparing of the temporal quadrant, may suggest M-type rather than P-type RGC injury [11]. Similar pattern loss is also observed in glaucoma, Alzheimer’s disease and Multiple System Atrophy [11]. Mechanisms for the possible selective damage to M-type RGC in GD1 requires further exploration. Functional visual evaluations in patients with GD1, such as electroretinogram (ERG), visual evoked potential (VEP) and perimetry (visual field) tests, can help assess RGC activity in these patients, as these tests correlate with RGC counts and activity [22,23,24].

There are several limitations to the present study. First, although we excluded patients with diagnosed glaucoma, a disease known to alter RNFL and inner retinal thickness, this exclusion was based on self-reporting and examination of medical records. Intraocular pressure and dilated fundus examinations were not included in the study protocol. It is possible that patients with undiagnosed glaucoma, were included. However, as glaucoma is not listed among the GD1-related comorbidities this limitation may affect both cohorts [15,25]. Second, although ND was ruled out by medical history and examination of medical records a full clinical assessment to diagnose ND was not performed. As patients with ND have previously been shown to have thinner macular GCC and pRNFL, and since some ND such as PD are more common in patients with GD, further studies that better exclude this population are necessary [11,26]. Last, refractive status was evaluated utilizing an autorefractometer and subjective refraction was not performed. The study population included individuals above 40 years of age, with limited accommodation ability and yet this may have resulted in less accurate refraction results. This limitation however would influence both cohorts as refractive errors are not considered to be associated with GD.

In conclusion, this study demonstrated that patients with GD1 exhibit pRNFL loss and macular GCC thinning compared to a healthy control group. Since the pattern of loss resembles that of glaucomatous damage, ophthalmologists examining patients with GD1 should be aware of the possibility of this alternative source of macular GCC and pRNFL damage. These findings add to the accumulating data implicating neurologic involvement in patients with GD1. Clinical follow-up of patients with GD1 with routine retinal OCT–may be important since retinal thinning may serve as an early biomarker for developing ND. Studies that examine ocular and visual pathway changes, as well as functional visual deficiencies, in patients with GD1 are of interest to define disease involvement and influence in the human eye.

## 4. Materials and Methods

This prospective case control study adhered to the Declaration of Helsinki and was approved by the Institutional Review Board of Shaare Zedek Medical Center (SZMC). Informed consent was obtained from all participants.

### 4.1. Eligibility Criteria

This prospective study included a cohort of 30 consecutive patients with GD1 followed at the SZMC Gaucher unit and 30 healthy controls. Participants between the ages of 40–75 years were included. Patients with a known ND and movement disorder, high myopia (−6.0 D>), previous intraocular surgeries apart from cataract extraction, glaucoma and poor-quality scans (quality index <30) were excluded from the study.

### 4.2. Study Design

Medical records were used to obtain patients’ demographics (age, gender), GD1 genotype, current treatment status, disease monitoring biomarker (glucosyl sphingosine (lyso-Gb1)) at the time of the retinal evaluation and disease severity score (Zimran severity score index (SSI)). lyso-Gb1 levels were measured as previously described, using liquid chromatography mass spectrometry of dried blood spot (DBS) samples (Centogene^®^, AG, Rostock, Germany) [27]. The Zimran SSI were recorded from the time of GD1 diagnosis [28]. Gaucher disease genotypes were divided to mild and severe (N370S/N370S being mild and N370S/IVS, N370S/84GG, N370S/203delc, N370S/L444P, N370S/R359X, N370S/del55p being severe) [29].

Autorefraction test without cycloplegia was performed on all patients. Volumetric macular and ON OCT scans of both eyes were performed without pupil dilation utilizing swept-source OCT (DRI OCT-1 Atlantis; version 9.30.003.02, Topcon, Tokyo, Japan). Optical coherence tomography scans were obtained by two experienced examiners (Y.W, Y.B). Retinal image quality indices were recorded for each OCT examination using the device’s automatic image quality value (quality index ranges from 0–100, manufacture’s recommendation ≥30). All OCT measurements were obtained using the built-in automatic segmentation incorporated in the Atlantis OCT device.

**Figure 2:**
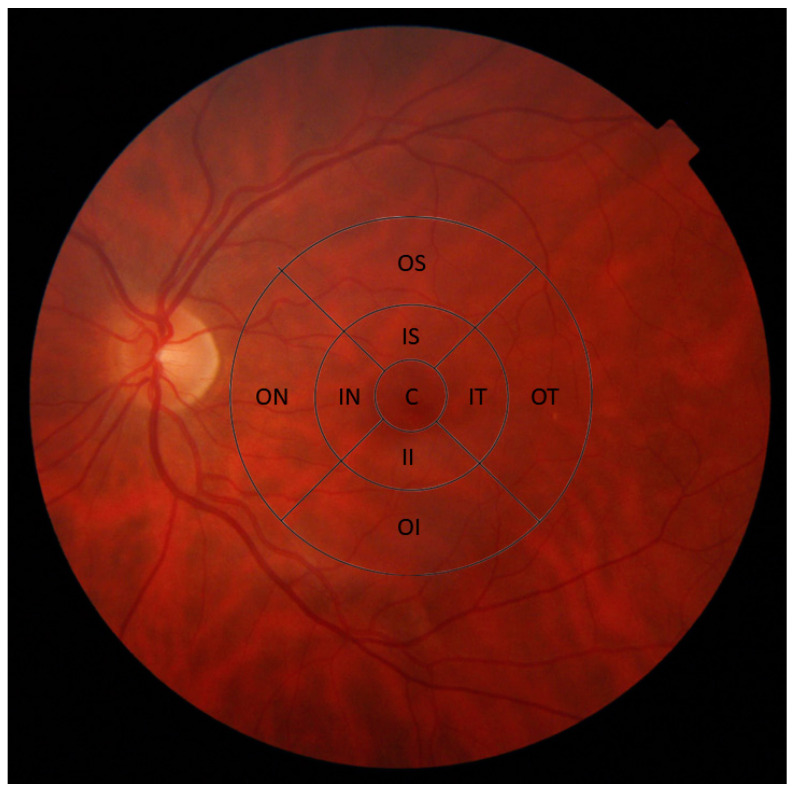
Early treatment diabetic retinopathy study (ETDRS) macular grid depicting nine macular sectors. Abbreviations: C—central; IS—inner superior; IT—inner temporal; II—inner inferior; IN—inner nasal; OS—outer superior; OT—outer temporal; OI—outer inferior; ON—outer nasal.

**Figure 3:**
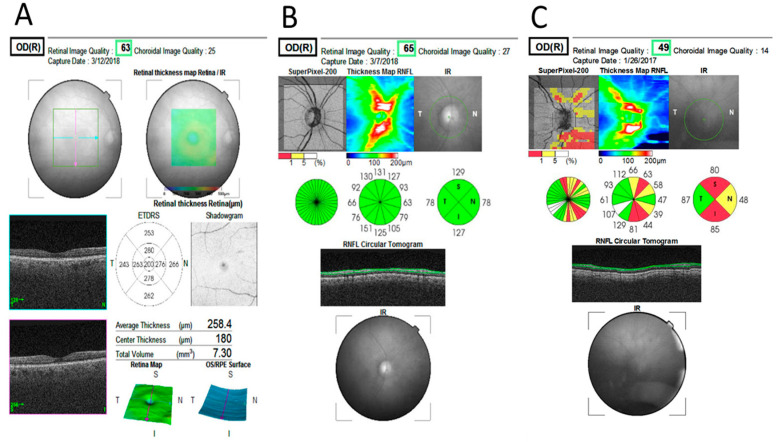
Examples of macular and optic nerve optical coherence tomography (OCT) printouts. (**A**) Normal macula with total retinal thickness displayed on the early treatment diabetic retinopathy study (ETDRS) map; (**B**) normal optic nerve retinal nerve fiber layer OCT-thickness map printout. Normal values (green colored) are displayed in all optic nerve quadrants; (**C**) abnormal optic nerve retinal nerve fiber layer OCT-thickness map printout displaying severe thinning in the superior and inferior quadrants (red) and moderate thinning in the nasal quadrant (yellow). Notice the relative perseverance of the temporal quadrant (green).

For macular evaluation, the 3D macula scan was used (automatic macular fixation, 6 mm × 6 mm, 512 × 216 (216 B-scans, each consisting of 512 A-scans)). The early treatment diabetic retinopathy study (ETDRS) criteria was employed in order to evaluate macular thickness in three concentric rings and was automatically adjusted to the center of the fovea (Figure 2) [30]. The first circle, of 1 mm diameter, represents the central macula. Surrounding it, a 3 mm-diameter ring represents the inner macula and a 6 mm-diameter ring represents the outer macula. The inner and outer rings were each segmented to nasal, temporal, superior and inferior quadrants. Macular GCC thickness (from the RNFL to the inner plexiform layer (IPL)–inner nuclear layer (INL) junction) total retina thickness (from the inner limiting membrane (ILM) until retinal pigment epithelium (RPE)) and macular volume were measured (Figure 1 and Figure 3).

For optic disc OCT assessment, the 3D optic disc scan was utilized (automatic disc fixation, 6 mm × 6 mm, 512 × 216 (216 B-scans, each consisting of 512 A-scans)). Peripapillary RNFL is defined by the device as the RNFL at the 3.4 mm circle automatically centered on the optic disc. The pRNFL map is automatically presented as a circle, which is divided to four, 12 and 36 sectors for analysis (Figure 3). For the study purpose, the built-in pRNFL quadrant map of each study participant was evaluated. The thickness of each quadrant (nasal, temporal, superior and inferior) was automatically displayed, as well as the total pRNFL thickness average. Each quadrant was displayed in one of three colors- green, yellow or red, indicating normal pRNFL thickness and thickness below the lower 5% and 1% of the population (adjusted to age), respectively (Figure 3). Peripapillary RNFL OCT scan was defined as abnormal if at least one quadrant was colored yellow or red.

### 4.3. Statistical Analysis

In order to test the association between two categorical variables, the chi-squared test was performed. Independent sample Mann–Whitney U Test was used to compare OCT data between patients with GD1 and controls and within patients with GD1 using the following variables: GD genotype and treatment status. The Pearson’s correlation coefficient was applied to observe the correlations between OCT data to GD disease monitoring marker (lyso-Gb1) and disease severity score (Zimran SSI). Due to multicomparison, a *p* value of ≤0.01 was considered statistically significant. Analyses were performed using the SPSS-software (SPSS 25.0; SPSS, Inc., Chicago, IL, USA).

## Figures and Tables

**Table 1:** Demographics and refraction of Gaucher disease patients and controls.

	Control Group	Gaucher Patients	*p* Value
Males (%)	13 (43.3%)	16 (53.3%)	0.492
Age (years)	51.17 ± 8.93	51.10 ± 9.79	0.729
Refraction (D)	−0.78 ± 2.14	−1.28 ± 2.30	0.542

D, Diopters.

**Table 2:** Quality and data of macular and optic nerve optical coherence tomography scans of eyes of patients with type-1 Gaucher disease and control group.

	Control Eyes (*n* = 60)	Gaucher eyes (*n* = 60)	*p* Value
Macular OCT quality	62.97 ± 5.17	61.28 ± 6.35	0.142
Macular volume (mm^3^)	7.69 ± 0.38	7.66 ± 0.27	0.648
Macular retinal thickness (µm)	271.98 ± 13.34	270.82 ± 9.62	0.631
Optic nerve OCT quality	62.62 ± 4.15	61.5 ± 5.02	0.196
Abnormal optic nerve scans (%)	5 (8.3%)	22 (36.7%)	**<0.001**
Peripapillary RNFL (µm)			
Average	106.40 ± 8.78	97.63 ± 8.42	**<0.001**
Temporal	73.63 ± 11.81	69.13 ± 13.40	0.220
Superior	130.57 ± 11.55	118.78 ± 16.15	**<0.001**
Nasal	83.30 ± 15.73	77.38 ± 22.04	0.015
Inferior	138.07 ± 18.09	125.20 ± 16.36	**<0.001**
Macular GCC layer (µm)			
Total average	73.92 ± 4.90	72.60 ± 4.84	0.557
Outer Average	64.89 ± 4.63	62.83 ± 4.69	0.226
central	47.70 ± 10.88	50.03 ± 10.34	0.110
inner temporal	86.70 ± 6.48	85.28 ± 7.64	0.417
Inner superior	91.07 ± 6.52	89.02 ± 8.38	0.250
Inner nasal	92.02 ± 7.24	89.77 ± 7.71	0.281
Inner inferior	88.28 ± 8.55	88.03 ± 7.51	0.646
Outer temporal	68.17 ± 5.49	66.65 ± 5.28	0.091
Outer superior	60.12 ± 4.63	59.58 ± 5.08	0.315
Outer nasal	68.02 ± 5.24	65.17 ± 6.17	**0.003**
outer inferior	63.92 ± 6.55	59.95 ± 6.50	**0.002**

Bold indicates significant. SD—standard deviation; OCT—optical coherence tomography; RNFL—retinal nerve fiber layer; GCC—ganglion cell complex.

## Supplementary Materials

Supplementary materials can be found at https://www.mdpi.com/1422-0067/21/19/7027/s1.

## Abbreviations

GD1	Type-1 Gaucher disease
pRNFL	Peripapillary retinal nerve fiber layer
OCT	Optical coherence tomography
GCC	Ganglion cell complex
ETDRS	Early treatment diabetic retinopathy study
GD	Gaucher disease
PD	Parkinson disease
RGC	Retinal ganglion cells
GCL	Ganglion cell layer
RNFL	Retinal nerve fiber layer
ON	Optic nerve
GD3	Type-3 Gaucher disease
ERG	Electroretinogram
VEP	Visual evoked potential
SZMC	Shaare Zedek Medical Center
DBS	Dried blood spot
IPL	Inner plexiform layer
INL	Inner nuclear layer
ILM	Inner limiting membrane
RPE	Retinal pigment epithelium
